# Standardized care pathway reshaped the diagnostic and therapeutic landscape of urinary bladder cancer. A 15‐year population‐based study

**DOI:** 10.1002/bco2.70179

**Published:** 2026-03-04

**Authors:** Mansour Istamulov, Hanna Eriksson, Suleiman Abuhasanein

**Affiliations:** ^1^ Department of Urology, Institute of Clinical Science, Sahlgrenska Academy University of Gothenburg Gothenburg Sweden; ^2^ Department of Surgery, Urology Section NU‐Hospital Group Uddevalla Region Västra Götaland Sweden; ^3^ Department of Research and Development NU‐Hospital Group Trollhättan Region Västra Götaland Sweden

**Keywords:** diagnostic delay, guideline adherence, healthcare delivery, haematuria, resection of tumour, standardized care pathway, survival analysis, treatment intervals, urinary bladder cancer

## Abstract

**Objectives:**

This work aimed to evaluate the long‐term impact of standardized care pathway (SCP) implementation for urinary bladder cancer (UBC) on tumour characteristics, diagnostic and treatment intervals and guideline adherence.

**Materials and Methods:**

A retrospective cohort study was conducted including all patients with newly diagnosed UBC at the NU Hospital Group, Sweden, between 2010 and 2024. Patients were grouped into pre‐SCP (2010–2015) and during‐SCP (2016–2024) cohorts. Patient demographics, tumour characteristics, adherence to guideline‐recommended care and diagnostic and treatment time intervals were analysed. Overall survival was assessed using Kaplan–Meier analysis and Cox proportional hazards regression.

**Results:**

Following SCP implementation, emergency presentations declined significantly (15% pre‐SCP to 10% SCP, *p* = 0.003). Tumour characteristics shifted towards earlier‐stage disease, with increased detection of small tumours ≤30 mm (56% to 71%, *p* < 0.001), fewer muscle‐invasive cases (27% to 21%, *p* = 0.003) and a higher proportion of TaG1–2 tumours (42% to 52%, *p* = 0.003). Adherence to guidelines improved markedly, reflected in cT1 disease by increased second‐look resections (36% to 69%, *p* < 0.001) and multidisciplinary team conference discussions (2% to 88%, *p* < 0.001). Diagnostic efficiency improved, with median referral‐to‐TURBT time reduced from 29 to 14 days (*p* < 0.001). In multivariable analysis, age, emergency admission, higher tumour stage and size and TURBT delay >18 days were independently associated with worse overall survival. Kaplan–Meier analysis revealed a temporal shift: Early rapid TURBT was associated with poorer survival in 2010–2015 but conferred a survival benefit in 2016–2024 (log‐rank *χ* ^2^ = 13.66, *p* = 0.003).

**Conclusions:**

SCP implementation was associated with earlier detection, improved guideline adherence and sustained reductions in diagnostic delays. However, increasing delays to definitive treatment for muscle‐invasive disease highlight emerging system‐level constraints, underscoring the need to optimize downstream capacity to fully realize the benefits of early diagnosis.

**Abbreviations**
CI: confidence interval
CTU: computed tomography urography
IQR: interquartile range
IVIT: intravesical instillation therapy
MDTC: multidisciplinary team conference
SCP: standardized care pathway
SLR: second‐look resection
TNM: tumour‐node‐metastasis
TURBT: transurethral resection of bladder tumour
UBC: urinary bladder cancer
WHO: World Health Organization

## 1 INTRODUCTION

Urinary bladder cancer (UBC) is a common global malignancy, predominantly affecting men.^1^ Delays in diagnosing or treating UBC are associated with worse outcomes.^2^, ^3^ System delays significantly contribute to total cancer delay, highlighting the need for shorter pathways, improved professional awareness, better diagnostics and targeted cancer‐specific interventions.^4^ Shorter referral delays may improve survival, although comorbidity can obscure these associations,^5^ and the reduced referral‐to‐investigation interval does not necessarily shorten investigation‐to‐treatment interval.^6^

To promote earlier diagnosis, proposed strategies include increased public awareness and improved education of primary care physicians to enhance patient assessment and guideline‐based referral.^7^ In Sweden, a standardized care pathway (SCP) for patients with suspected UBC was implemented nationwide in 2016 mandated immediate referral for macroscopic haematuria, rapid diagnostics with cystoscopy and computed tomography urography (CTU) and timely transurethral resection of bladder tumour (TURBT).^8^ Despite clear time frames, national implementation initially showed regional variation in adherence.^9^

Following the introduction of the SCP, the national median time from referral to TURBT in Sweden decreased from 37 to 27 days.^9^ At an institution where SCP guidelines were strictly followed, improvements were greater: median time to TURBT fell from 29 to 12 days (the recommended time frame was 13 days), with a decrease in muscle‐invasive tumours (MIBC) from 26% to 20%.^10^ Although the SCP initially was associated with faster diagnostics and improvements in tumour characteristics, long‐term SCP sustainability remains uncertain. Given these uncertainties and to assess whether early SCP gains have persisted, this study aims to examine tumour characteristics, time‐intervals and guideline adherence in UBC patients nine years after SCP implementation.

## 2 MATERIALS AND METHODS

### 2.1 Study design and data

This is a retrospective cohort study conducted at the NU Hospital Group, a regional healthcare provider in Western Sweden serving approximately 320 000 inhabitants. The study included all patients with newly diagnosed UBC (ICD‐10: C67) between 1 January 2010 and 31 December 2024. Patients were managed according to local clinical routines before 2016 and according to the SCP for suspected UBC after that.^8^ To see changes that were provided by SCP but also how those changes developed during this time, the study period was divided into three cohorts, the pre‐SCP period (2010–2015), early‐SCP period (2016–2019) and late‐SCP period (2020–2024). Early‐ and late‐SCP cohorts were combined into a general SCP period for the main analysis and presentation unless otherwise stated or shown. Patients with a new histopathological diagnosis of UBC between 1 January 2010 and 31 December 2024 were eligible for inclusion in the respective cohort, and the rest were excluded.

Data for the earlier cohorts (2010–2019) were extracted from previously established datasets used in an earlier published study^10^ with follow‐up updated through 30th September 2025, whereas data for the late‐SCP cohort (2020–2024) were obtained through manual review of electronic medical records using the same methodology as in the earlier periods. All data were compiled into a predefined spreadsheet and included patient characteristics such as age, sex, admission modality (referral, emergency admission or others) and reason for investigation (categorized as either macroscopic haematuria or other indications).

Tumour characteristics were registered according to the same protocol across all study years, including clinical tumour‐node‐metastasis (TNM) stage (classified according to the TNM 8th edition^11^) and tumour grade (assigned according to the WHO/ISUP 1999 system^12^). Tumour size was defined as the largest measured diameter in millimetres, and multiplicity was documented as single or multiple lesions. Between two or more pathological grades, the higher one was recorded. Also, diagnostic and treatment variables included the time intervals from referral to first urological assessment, from referral to TURBT, from TURBT to second‐look resection (SLR), to the intravesical instillation therapy (IVIT), to multidisciplinary team conference (MDTC) or to cystectomy. Eligibility for IVIT included large (≥30 mm) and/or multiple TaG1–2 tumours, all TaG3, T1 or carcinoma in situ. A single postoperative instillation was not utilized at our institution. Eligibility for SLR included all cT1 tumors.

### 2.2 Standardized care pathway

The SCP introduced a structured outpatient workflow to streamline management and diagnostics, including all patients aged ≥50 with macroscopic haematuria or those with radiological findings suggestive of UBC, regardless of the imaging's original indication. To support this, an educational campaign was directed at primary care physicians to emphasize the importance of prompt evaluation of all patients with suspected UBC, most commonly presenting with macroscopic haematuria.^8^, ^13^ Patients who meet the criteria should be referred from primary or other healthcare services within 24 h. Following referral, they were initially intended to undergo a CTU and cystoscopy within 7 days. The pathway also stipulates that TURBT should be performed as soon as possible. While the initial time limit for TURBT was 13 days, this was revised in 2024 to a new target of 18 days.^8^ Furthermore, all patients diagnosed with stage cT1 should undergo a SLR, and those with cT1+ should be discussed at MDTC.

### 2.3 Statistics

Continuous variables were summarized using median and interquartile ranges (IQRs), whereas categorical variables were presented as frequencies and percentages. Comparisons between groups were conducted using the chi‐square test for categorical variables and the Wilcoxon rank‐sum test for continuous variables. To assess clinical prioritization and adherence to SCP time targets, the interval from referral to TURBT was dichotomized using the new Swedish guideline threshold, applying a cut‐off of ≤18 days for all study periods.

To examine prognostic factors for patient outcomes, we performed univariable and multivariable Cox proportional hazards regression analyses. In this analysis, patients followed until death, end of follow‐up or 30th September 2025, whichever came first. However, survival time was censored at 60 months to reduce the large discrepancy between patients diagnosed in 2010 and those diagnosed in 2024. Age, admission modality, 18 days cut off (referral to TURBT), tumour size, tumour multiplicity and tumour stage were included as explanatory variables. Results from the Cox models are presented as hazard ratios (HR) with corresponding 95% CI.

Differences in referral‐to‐TURBT time intervals across admission modalities and age groups were assessed using the Kruskal–Wallis test. Annual trends (2010–2024) in median time intervals for diagnostic and treatment milestones were shown with line graphs, for all patients and for those with T2+ disease. Overall survival was compared across pre‐SCP and SCP cohorts, stratified by referral‐to‐TURBT time (≤18 vs. >18 days) using log‐rank tests. *p* values <0.05 were considered statistically significant. Statistical analyses were performed using SPSS Statistics version 30 (IBM Corp., Armonk, NY, USA).

## 3 RESULTS

A total of 1239 patients with newly diagnosed UBC were identified during 2010–2024. The median age was 76 years (IQR 69–81), and 22% (277) of all patients were women (Table 1). Emergency presentations significantly decreased over the study period (15% pre‐SCP to 10% during SCP, *p* = 0.003). Looking more specifically at all three periods, a declining trend can be seen from 15% pre‐SCP, 13% early‐SCP to 7% late‐SCP (*p* < 0.001) (Table 2).

**TABLE 1:** Descriptive parameters of all patients diagnosed with urinary bladder cancer in the NU Hospital Group between 2010 and 2024 stratified into two time periods in relation to the implementation of SCP. Figures represent the number of patients (% of the column) if not otherwise indicated (IQR: interquartile range, SCP: standardized care pathway, TURBT: transurethral resection of tumour in urinary bladder, IVIT: intravesical instillation therapy, SLR: second look resection, MDTC: multidisciplinary team conference, RT: radiation therapy).

Variable name		Pre‐SCP 2010–2015	SCP 2016–2024	All	*p* value
No. patients	(% of the row)	455 (37)	784 (63)	1239	
Gender, *n* (%)	Male	357 (79)	605 (77)	962 (78)	0.598
	Female	98 (22)	179 (23)	277 (22)	
Age (years)	Median, (IQR)	75 (67–81)	76(70–82)	76 (69–81)	0.09
Admission modality	Referral	371 (82)	664 (85)	1035 (84)	0.003
	Emergency	69 (15)	75 (10)	144(12)	
	Others^a^	15 (3)	45 (6)	50 (5)	
Reason for investigation	Macroscopic haematuria	350 (77)	608 (78)	958 (77)	<0.001
	LUTS	56 (12)	48 (6)	104 (8)	
	Others^b^	49 (11)	128 (16)	177 (14)	
Radiology	For diagnosis	408 (90)	749 (96)	1157 (93)	<0.001
	Image‐suspected tumour	247 (54)	571 (73)	818 (66)	<0.001
Number of tumours^c^	Single	294 (68)	489 (63)	783 (65)	0.110
	Multiple	141 (32)	287 (37)	428 (35)	
Tumour size^c^	≤30 mm	169 (56)	512 (71)	681 (67)	<0.001
	>30 mm	131 (44)	212 (29)	343 (34)	
Tumour grade, *n* (%)	G1	58 (13)	84 (11)	142 (12)	0.002
	G2	257 (57)	381 (49)	638 (52)	
	G3	140 (31)	319 (41)	459 (37)	
T, *n* (%)	TaG1–2^d^	191 (42)	407 (52)	598 (48)	0.003
	TaG3, Tis, T1	141 (31)	211 (27)	352 (28)	
	T2+	123 (27)	166 (21)	289 (23)	
N, *n* (%)	N+	7 (2)	32 (4)	39 (3)	0.013
M, *n* (%)	M1	9(2)	24 (3)	33 (3)	0.254
IVIT, *n* (%)	For eligible patients^e^	141 (65)	251(65)	392 (65)	0.857
SLR, *n* (%)	For T1^f^	46 (36)	103(69)	149 (54)	<0.001
MDTC, *n* (%)	For T1+^f^	4 (2)	278 (88)	282 (50)	<0.001
Cystectomy, *n* (%)	For T2+^f^	64 (52)	66 (40)	130 (45)	0.038
RT			18	18	
Time to TURBT (days)	Median, (IQR)	29 (16–48)	14 (9–24)	19 (11–34)	<0.001
Time to TURBT, *n* (%)	≤ 18 days	122 (27)	497 (63)	619 (36)	<0.001
	>18 days	333 (73)	287 (37)	720 (64)	

^a^ “Others” includes admission for lower urinary tract symptoms, urinary tract infection, etc.

^b^ “Others” includes incidental findings, patient‐reported symptoms, perioperative findings and other nonhaematuria indications.

^c^ Missing samples for tumour size: 155 in 2010–2015, 60 in 2016–2019 and 0 in 2020–2024.

^d^ TaG1–2 tested against all other T categories together; TaG3, Tis and T1 tested against all other T categories together; T2+ tested against all other T categories together.

^e^ Eligibility for IVIT defined: TaG1–2 with multiplicity and/or tumour size >3 cm, TaG3, Tis or T1.

^f^ Patients within timeframes included in corresponding analyses: SLR—6 months, MDTC—9 months and cystectomy—14 months.

**TABLE 2:** Descriptive parameters of all patients diagnosed with urinary bladder cancer in the NU Hospital Group between 2010 and 2024 stratified into three time periods in relation to the implementation of SCP. Figures represent the number of patients (% of the column) if not otherwise indicated (IQR: interquartile range, SCP: standardized care pathway, TURBT: Transurethral resection of tumour in urinary bladder, IVIT: intra‐vesical instillation therapy, SLR: second look resection, MDTC: multidisciplinary team conference, RT: radiation therapy).

Variable name		Pre SCP 2010–2015	Early SCP 2016–2019	Late SCP 2020–2024	All	*p* value
No. patients	(% of the row)	455 (37)	330 (27)	454 (37)	1239 (100)	
Gender, *n* (%)	Male	357 (79)	245 (74)	360 (79)	962 (78)	0.214
	Female	98 (22)	85 (26)	94 (21)	277 (22)	
Age (years)	Median, (IQR)	74 (67–81)	75 (70–82)	75 (69–81)	76 (69–81)	0.237
Admission modality	Referral	371 (82)	273 (83)	391 (86)	1018 (82)	<0.001
	Emergency	69 (15)	43 (13)	32 (7)	141(11)	
	Others^a^	15 (3)	14 (4)	31 (7)	80 (7)	
Reason for investigation	Macroscopic haematuria	350 (77)	259 (78)	349 (77)	958 (77)	0.840
	Others^b^	105 (23)	71 (22)	105 (23)	281 (23)	
Radiology	Before TURBT	408 (90)	320 (97)	429 (95)	1157 (93)	
	Image‐suspected tumour	245 (60)	243 (76)	328 (76)	816 (71)	
Number of tumours^c^	Single	306 (67)	200 (61)	292 (64)	798 (64)	0.186
	Multiple	149 (33)	130 (39)	162 (36)	441 (36)	
Tumour size^c^	≤30 mm	169 (56)	181 (67)	331 (73)	681 (67)	<0.001
	>30 mm	131 (44)	89 (33)	123 (27)	343 (33)	
Tumour grade, *n* (%)	G1	58 (13)	40 (12)	44 (10)	142 (12)	0.010
	G2	257 (57)	158 (48)	223 (49)	638 (52)	
	G3	140 (31)	132 (40)	187 (41)	459 (37)	
cT, *n* (%)	TaG1–2^d^	191 (42)	162 (49)	245 (54)	598 (48)	0.001
	TaG3, Tis, T1	141 (31)	92 (28)	119 (26)	352 (29)	0.271
	T2+	123 (27)	76 (23)	90 (20)	289 (23)	0.036
cN, *n* (%)	N+	7 (2)	10 (3)	22 (5)	40 (3)	0.017
cM, *n* (%)	M1	9(2)	8 (2)	16 (4)	28 (2)	0.334
IVIT, *n* (%)	For eligible patients^e^	141 (65)	106 (62)	145 (64)	392 (65)	0.857
SLR, *n* (%)	For cT1^f^	46 (36)	50 (69)	53 (70)	149 (54)	<0.001
MDTC, *n* (%)	For cT1+^f^	4 (2)	132 (89)	146 (88)	282 (50)	<0.001
Cystectomy, *n* (%)	For cT2+^f^	64 (52)	37 (49)	29 (32)	130 (45)	0.012
RT				18 (20)	18	
Time to TURBT (days)	Median, (IQR)	29 (16–48)	12 (8–19)	15 (11–27)	19 (11–34)	<0.001
Time to TURBT, *n* (%)	0–18 days	122 (27)	242 (73)	255 (56)	447 (36)	<0.001
	>18 days	333 (73)	88 (27)	199 (44)	793 (64)	

^a^ “Others” includes lower urinary tract symptom, urinary tract infection and so forth.

^b^ “Others” includes incidental findings, patient‐reported symptoms, perioperative findings and other nonhaematuria indications.

^c^ Missing samples for tumour size: 155 in 2010–2015, 60 in 2016–2019 and 0 in 2020–2024.

^d^ TaG1–2 tested against all other T categories together; TaG3, TIS and T1 tested against all other T categories together; T2+ tested against all other T categories together.

^e^ Eligibility for IVIT defined: TaG1–2 with multiplicity and/or tumour size >3 cm, TaG3, Tis or T1.

^f^ Patients within timeframes included in corresponding analyses: SLR—6 months, MDTC—9 months and cystectomy—14 months.

A significant shift towards diagnosing smaller tumours (≤30 mm) was observed, rising from 56% pre‐SCP to 71% during SCP (*p* < 0.001). The proportion of MIBC decreased from 27% to 21% (*p* = 0.003). Conversely, low‐grade tumours (TaG1–2) increased from 42% to 52% (*p* = 0.003) (Table 1), with a progressive rise across periods: 42% pre‐SCP, 49% early‐SCP and 54% late‐SCP (*p* = 0.001) (Table 2). Following SCP implementation, SLR for cT1 disease rose substantially (36% to 69%, *p* < 0.001), as did MDTC discussions (2% to 88%, *p* < 0.001). Radiological examination was also used more often, going from 90% to 96% during SCP (*p* < 0.001) (Table 1).

**FIGURE 1:**
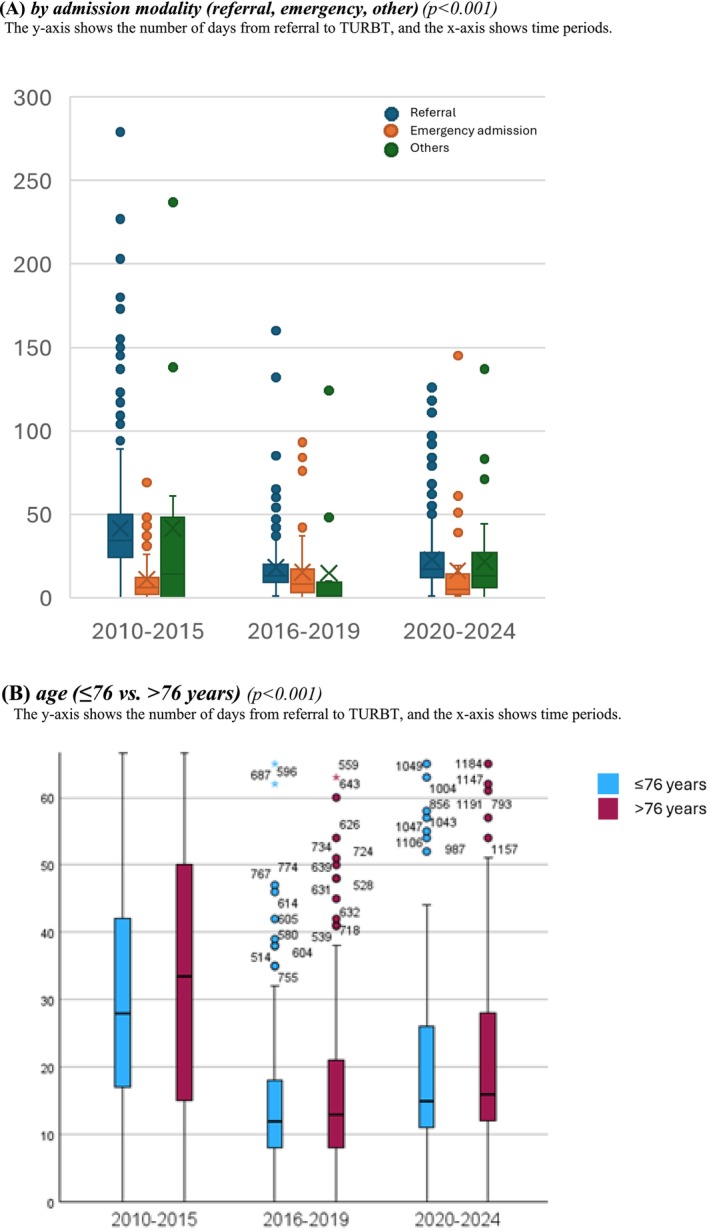
Time trends in referral‐to‐TURB intervals across study periods in all patients diagnosed with urinary bladder cancer at the NU Hospital Group between 2010 and 2024, stratified.

**FIGURE 2:**
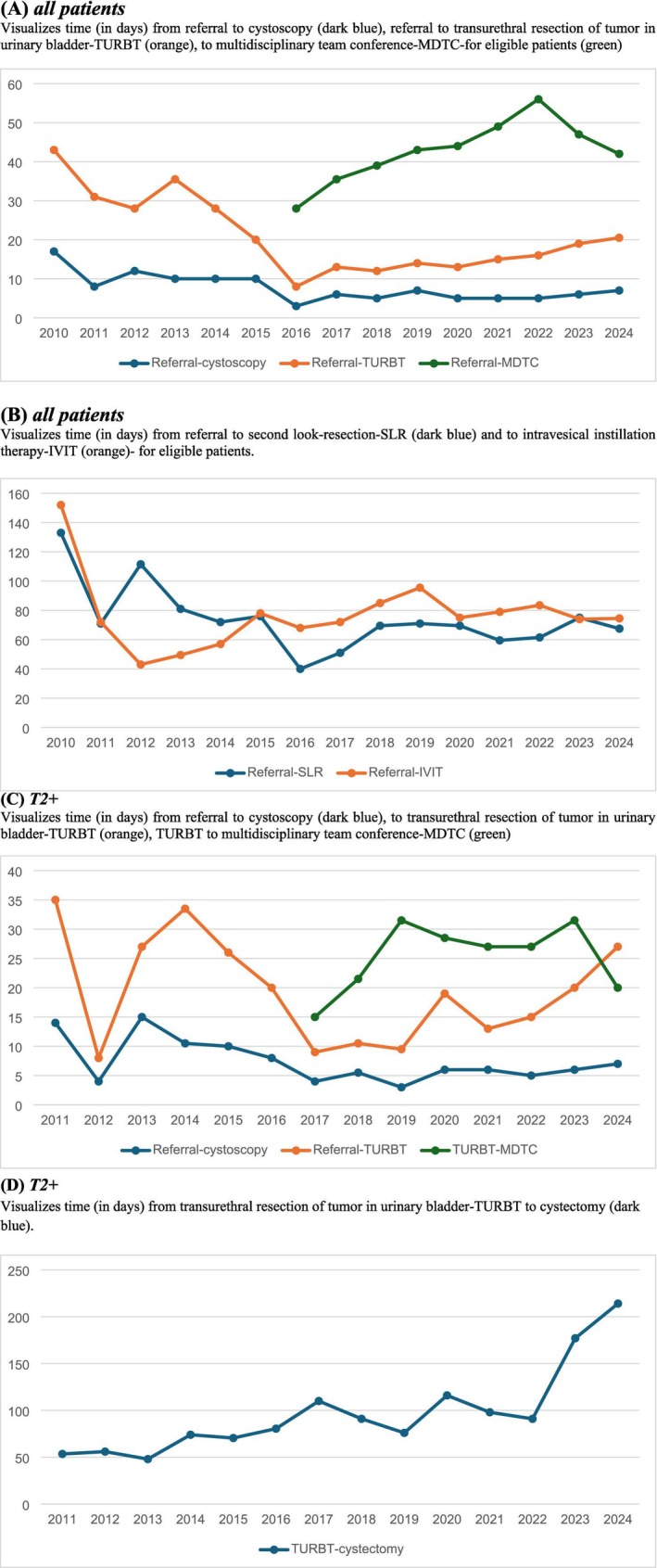
Annual time intervals across key steps in the urinary bladder cancer diagnostic and treatment pathway at the NU Hospital Group (2010–2024).

We observed an increased proportion of patients undergoing TURBT within 18 days (27% to 63%, *p* < 0.001), and subsequently the median time to TURBT also fell significantly, from 29 days pre‐SCP to 14 days during SCP (*p* < 0.001) demonstrating a clear decline in delays across periods. Differences by admission modality remained, with emergency patients consistently experiencing the shortest waiting times (*p* < 0.001). Age‐stratified analyses showed longer delays among patients aged ≥76 years compared with younger individuals (*p* < 0.001) (Figure 1a,b). Annual trends indicate shorter delays to TURBT, whereas MDTC and subsequent treatments show variability. Yearly fluctuations in TURBT, cystectomy, SLR and radiotherapy reflect capacity and case‐mix changes. In T2+ disease, key time intervals fluctuated, however, TURBT‐to‐cystectomy times rose steadily (Figure 2a–d).

In the multivariate Cox proportional hazards model for overall survival, several factors were associated with increased mortality (Table 3). Older age predicted worse survival (HR 1.05, 95% CI 1.04–1.06, *p* < 0.001). Emergency admission showed a strong association with higher mortality (HR 1.97, 95% CI 1.45–2.69, *p* < 0.001). Higher stages, including TaG3/T1/Tis (HR 1.50, 95% CI 1.10–2.03, *p* = 0.010) and particularly T2+ (HR 3.52, 95% CI 2.62–4.73, *p* < 0.001), were significant predictors. A treatment delay >18 days to TURBT was also linked to poorer survival (HR 1.34, 95% CI 1.05–1.72, *p* = 0.018).

**TABLE 3:** Multivariate and univariate Cox proportional hazards regression analyses performed on all patients with urinary bladder cancer in NU Hospital Group. Patients were followed from diagnosis until death, end of follow‐up or 30th September 2025, whichever occurred first. Patients were censored at 60 months to account for differences in follow‐up duration. (CI: confidence interval, HR: hazard ratio, SCP: standardized care pathway, TNM: tumour, lymph node, metastasis, TURBT: transurethral resection of bladder tumour).

Variable name	Multivariate HR (95%CI)	*p* value	Univariate HR (95%CI)	*p* value
Age	1.05 (1.04–1.06)	<0.001	1.06 (1.05–1.07)	0.296
Sex (male)	1		1	
Female	1.09 (0.84–1.43)	0.511	1.13 (0.90–1.41)	0.296
Admission modality (referral)	1		1	
Emergency vs. referral	1.97 (1.45–2.69)	<0.001	2.84 (2.25–3.57)	<0.001
Reason for investigation (haematuria)	1		1	
LUTS vs. haematuria	0.99 (0.66–1.47)	0.943	1.00 (0.67–1.50)	0.994
Number of tumours (single)	1		1	
Multiple	0.91 (0.71–1.17)	0.456	0.75 (0.60–0.92)	0.006
Tumour size (≤30 mm)	1		1	
> 30 mm	1.43 (1.11–1.84)	0.006	2.48 (1.99–3.10)	<0.001
T stage (TaG1–2)	1		1	
TaG3/T1/Tis vs. TaG1–2	1.50 (1.10–2.03)	0.010	1.68 (1.31–2.16)	<0.001
T2+ vs. TaG1–2	3.52 (2.62–4.73)	<0.001	4.10 (3.26–5.16)	<0.001
Time to TURBT, (≤18 days)	1		1	
>18 days	1.34 (1.05–1.72)	0.018	1.09 (0.90–1.33)	<0.001

**FIGURE 3:**
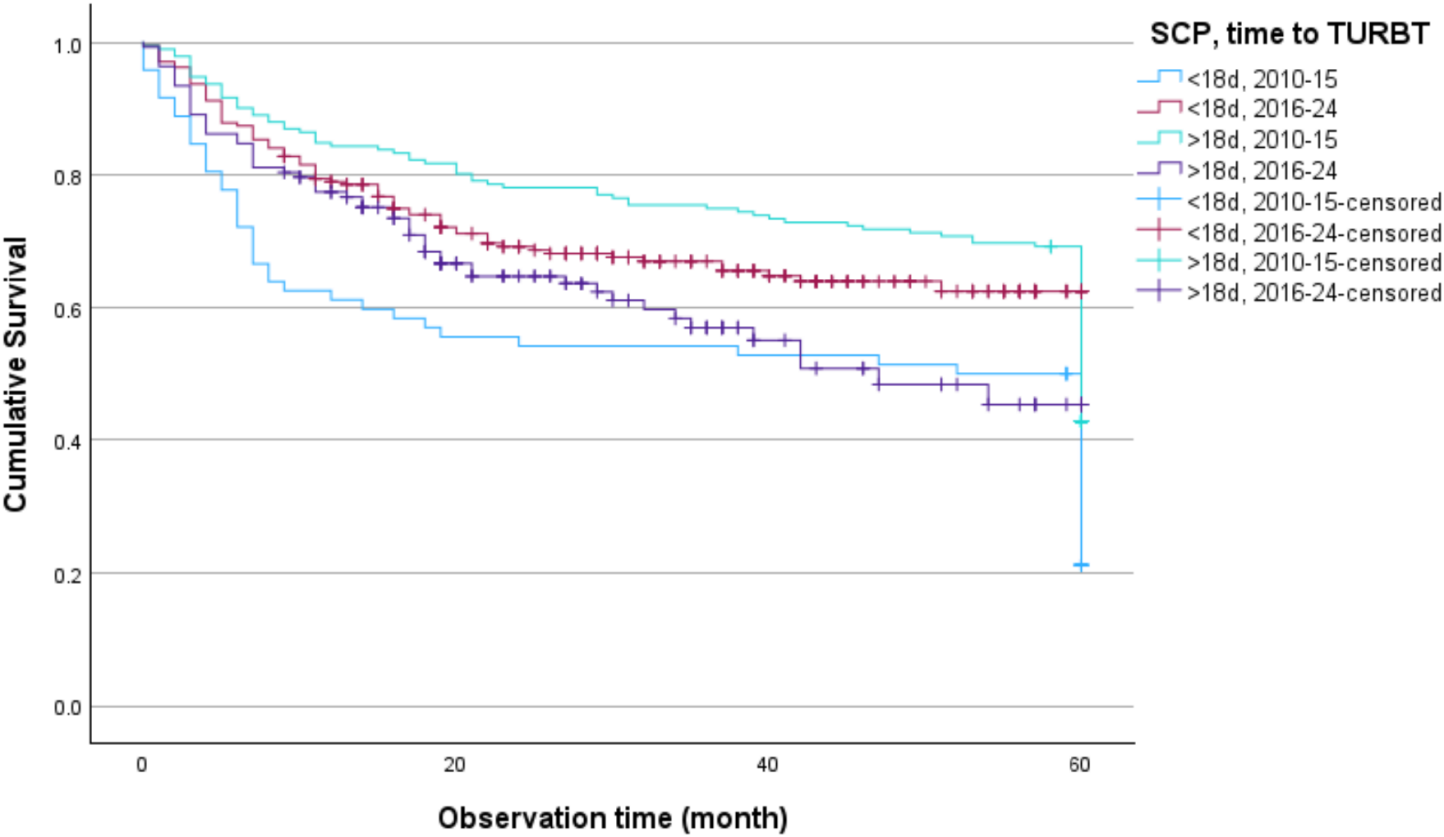
Kaplan–Meier curves of overall survival for all patients with UBC—except those with TaG1–2—(*n* = 641) at NU‐Hospital Group stratified into four groups according to time to TURBT (0–18 and >18 days) and before SCP 2010–2015 and during SCP 2016–2024 (log‐rank *χ* ^2^ = 13.66, *p* = 0.003). Difference between the groups 0–18 days before and after SCP: log‐rank *χ* ^2^ (10.86), *p* < 0.001. Difference between the groups >18 days before and after SCP: log‐rank *χ* ^2^ (8.32), *p* = 0.004.

Several characteristics differed between patients treated within ≤18 days and those treated later (Table S1). Kaplan–Meier analysis showed that in 2010–2015, treatment within 18 days was associated with poorer survival, whereas in 2016–2024, treatment within 18 days was linked to improved survival and delays beyond 18 days were unfavourable (log‐rank *χ* ^2^ = 13.66, *p* = 0.003) (Figure 3).

## 4 DISCUSSION

This study of 1239 patients with newly diagnosed UBC between 2010 and 2024 identified three key developments associated with the implementation of SCP. First, patient and tumour characteristics shifted towards earlier detection, with smaller tumours, fewer muscle‐invasive cases, more TaG1–2 disease and substantially fewer emergency presentations. Second, adherence to prevailing guidelines improved markedly, reflected by expanded use of SLR for cT1 tumours, a sharp increase in MDTC participation, and stronger compliance with recommended care processes. Third, the diagnostic‐time efficiency improved, with significantly shortened time to TURBT and better adherence to the recommended timelines. Survival analysis revealed a temporal shift: early rapid TURBT was associated with poorer survival in pre‐SCP 2010–2015 but conferred a survival benefit during SCP 2016–2024. In contrast, the timeframe for muscle‐invasive disease worsened, as TURBT‐to‐cystectomy intervals increased over time, indicating emerging bottlenecks in definitive treatment despite gains in early diagnostic performance.

### 4.1 Patient and tumour characteristics

Our study showed notable shifts in presentation patterns and tumour characteristics, most prominently a marked decline in emergency admissions. This indicates that more patients now enter care through planned referrals, likely due to improved referral practices and greater primary‐care awareness. The general practice role in early cancer detection by managing patient symptoms, recognizing cancer patterns and making appropriate referrals is central to this process.^14^ These trends mirror evidence that macroscopic haematuria warrants urgent evaluation and that fast‐track pathways enhance timely diagnostics.^15^

Parallel to this, the proportion of small tumours (≤30 mm) rose markedly, indicating that UBC is being detected at an earlier stage. This aligns with findings from a study of 169 patients, which showed that diagnostic delay is significantly associated with larger tumour size, and each day of delay increased pathological tumour size by approximately 0.088 mm.^16^ Given that tumour size influences risk stratification and subsequent management, these data reinforce the importance of timely detection and may help explain the observed shift towards smaller, earlier‐stage tumours. However, in our study, the pre‐SCP period had a lot of missing values, which could have been smaller tumours. Nevertheless, this aligns with previous findings showing that SCP was associated with earlier evaluation and higher detection rates of less advanced diseases.^10^

Tumour stage distribution also shifted notably over time, with a clear reduction in muscle‐invasive disease and an increase in low‐grade Ta tumours following SCP implementation. When compared with data from the Swedish National Registry for Bladder and Urinary Tract Cancer (SNRUBC), it is evident that our institution diagnoses a higher proportion of TaG1–2 tumours—54% (2020–2024) versus the national 42% (6442 of 15 271 UBC cases, 2020–2024).^17^ This difference may reflect the fact that many other hospitals have not fully implemented the SCP as intended, whereas adherence at our institution appears stricter. Overall, these patterns suggest that consistent application of the SCP can meaningfully reshape diagnostic pathways and contribute to earlier‐stage detection.

Despite improved referral practices, several clinical markers remain closely linked to poorer outcomes. Emergency admission continued to predict worse prognosis, likely reflecting later presentation and more advanced disease. Macroscopic haematuria presenting in the emergency department poses a significant challenge; in the UK, it represents 15% of all urological emergencies, with more than 25 000 admissions each year and prolonged hospital stays.^18^ Prior studies show that diagnostic delay increases UBC mortality, reinforcing our finding that delayed presentation—and thus emergency evaluation—is a key driver of poor outcomes.^19^

In our study, emergency admissions were reduced by approximately half, declining from 15% pre‐SCP to 7% in the late‐SCP period (*p* < 0.001). This reduction is important, as it indicates that more patients are being investigated through planned referrals, where assessment can be conducted more thoroughly and calmly. The IDENTIFY study further highlights the stakes: 32% of patients admitted acutely with haematuria had an underlying malignancy.^20^ Together, these findings underscore the need for timely outpatient evaluation pathways to ensure early cancer detection and avoid unnecessary emergency presentations.

### 4.2 Adherence to prevailing guidelines

We also observed improvements in several care parameters, including MDTC discussion for up to 88% of all T1+ patients. A systematic review of 51 studies showed that MDTCs improve diagnostic accuracy, treatment decision‐making and survival across multiple cancers.^21^ Our rising MDTC inclusion, therefore, likely contributes to more consistent staging, better‐informed management and enhanced quality of enhanced care, reinforcing the value of structured, standardized multidisciplinary workflows.

Furthermore, and following SCP implementation, the SLR rate for cT1 disease doubled, increasing from 36% pre‐SCP to 70% in the late‐SCP period (*p* < 0.001). This highlights both the value of SLR in this tumour group and strong guideline adherence. A large multicentre study of 2451 T1 high‐grade cases similarly demonstrated that SLR improves recurrence, progression and survival outcomes.^22^ Thus, our rising SLR rates likely reflect higher‐quality primary resections and more appropriate, evidence‐based use of re‐resection.

### 4.3 Timeframes in UBC care

While previous studies^9^, ^10^ documented only the initial decline in waiting times, our findings show that this improvement persisted through 2024, indicating that the SCP has become embedded in routine practice rather than representing a short‐term implementation effect. The Kaplan–Meier analysis demonstrated a clear temporal shift in the prognostic impact of time to TURBT. During 2010–2015, treatment within 18 days was associated with poorer survival, likely reflecting confounding by acute, high‐risk presentations. In contrast, during 2016–2024, timely TURBT within 18 days conferred a survival advantage, while delays beyond 18 days were unfavourable (log‐rank *χ* ^2^ = 13.66, *p* = 0.003). These findings align with time‐trend diagrams showing improved pathway efficiency over time.

Previous studies suggest that timely diagnosis and treatment of advanced bladder cancer may improve overall survival in selected patient groups.^23^ In our study, distinct patterns were observed across SCP intervals beyond time to TURBT. Referral‐to‐first‐visit times remained consistently short, indicating preserved access to initial evaluation. In contrast, intervals to SLR and additional procedures showed greater year‐to‐year variability, likely reflecting fluctuations in surgical capacity. Referral‐to‐MDTC intervals increased after 2016, consistent with expanded multidisciplinary involvement. Overall, these findings suggest that later treatment steps are more vulnerable to systemic pressures than early diagnostic milestones.

It is well established that delayed diagnosis is linked to poorer outcomes. However, patients who present late are often already vulnerable—they may have multiple comorbidities, require cardiological and anaesthesiologic assessment before surgery, or need time to optimize their general condition. These factors make them inherently frailer, introducing significant selection bias. This observation is consistent with the findings of Boeri et al.^24^ who reported that patients experiencing longer treatment delays were generally more medically compromised, and that their pre‐existing limitations—rather than the delay itself—likely contributed to poorer survival outcomes.

In our study, the steadily increasing TURBT‐to‐cystectomy interval may partly reflect healthcare centralization towards high‐volume centres. Evidence from large observational cohorts shows that hospitals performing ≥10–20 radical cystectomies annually achieve superior perioperative outcomes, supporting referral to specialized centres.^25^ While centralization likely improves survival and safety, it may introduce logistical delays due to limited capacity and referral pathways. Additionally, more extensive preoperative investigation with ^18^F‐fluorodeoxyglucose positron emission tomography combined with computed tomography (FDG‐PET‐CT) may contribute to longer TURBT‐to‐cystectomy intervals. Although FDG‐PET‐CT provides superior sensitivity and positive predictive value over conventional CT for lymph node staging in UBC, improving preoperative accuracy and prognostic stratification,^26^ its use may introduce additional delays before definitive surgery. Another contributing factor is the increasing use of neoadjuvant therapy before cystectomy. Although exact figures for the entire period are unavailable, its use has clearly risen, inevitably prolonging the interval from TURBT to cystectomy. This delay should not necessarily be viewed as negative because neoadjuvant chemotherapy improves survival and pathological response,^27^ indicating that the delay may reflect a deliberate, beneficial treatment strategy.

This study has several strengths. It includes a large, population‐based cohort spanning 15 years, capturing all newly diagnosed UBC cases within a clearly defined healthcare institution. The implementation of the SCP can therefore be evaluated in a real‐world clinical setting with high external validity. The division into pre‐, early‐ and late‐SCP periods allows for the assessment of temporal trends and maturing adherence over time. Another strength is the detailed granularity of clinical data, including tumour stage, grade, size, multiplicity and key time intervals along the diagnostic and treatment pathway.

However, several limitations must be acknowledged. The retrospective design makes the study vulnerable to missing data, unmeasured confounding and documentation variability, particularly in the early years. Changes in practice unrelated to the SCP—such as evolving TURBT techniques, improved imaging or shifting resource availability—may also contribute to observed trends. Moreover, the use of overall survival rather than cancer‐specific survival means that the findings may be influenced by non‐cancer deaths in this older cohort. Furthermore, short follow‐up for the most recent patients reduces the ability to detect long‐term survival differences. Finally, although the study examines pathway performance, it is not powered to detect survival differences, and follow‐up time for the late‐SCP cohort remained relatively short.

## 5 CONCLUSION

In conclusion, implementation of the SCP was associated with earlier detection of UBC, improved adherence to guideline‐recommended care and sustained reductions in diagnostic delays, particularly time to TURBT. These improvements coincided with shifts towards less advanced disease at diagnosis and a higher proportion of patients managed according to guidelines. However, increasing delays in definitive treatment for muscle‐invasive disease highlight emerging system‐level constraints. Continued optimization of downstream capacity is needed to ensure that gains in early diagnosis translate into timely, high‐quality treatment and improved outcomes.

## AUTHOR CONTRIBUTIONS

Conceptualization and design were performed by Suleiman Abuhasanein. Acquisition of data was carried out by Mansour Istamulov and Hanna Eriksson. Statistical analysis and interpretation of the data were conducted by Suleiman Abuhasanein and Mansour Istamulov. Drafting of the manuscript and critical revision of the manuscript for important intellectual content were completed by Suleiman Abuhasanein, Mansour Istamulov and Hanna Eriksson. Project administration, obtaining funding and supervision were performed by Suleiman Abuhasanein. All authors have approved the submitted version of the manuscript.

## CONFLICT OF INTEREST STATEMENT

The authors have no conflict of interest to declare.

## Supporting information


**Table S1.** Descriptive parameters of all patients with urinary bladder cancer in NU Hospital Group between 2010 and 2024 stratified into groups regarding to time to TURBT and the relation to the implementation of SCP. Figures represent number of patients (% of the column) if not otherwise indicated. (IQR: interquartile range, SCP: standardized care pathway, TURB: Transurethral resection of tumour in urinary bladder, IVIT: intra‐vesical instillation therapy, SLR: second look resection, MDTC: multidisciplinary team conference, RT: radiation therapy)

## Data Availability

The datasets used and/or analysed during the current study are available from the corresponding author on reasonable request.
