# Supplementary material for: Standardized care pathway reshaped the diagnostic and therapeutic landscape of urinary bladder cancer. A 15‐year population‐based study
Source: BJUI Compass. 2026 Mar 4;7(3):e70179. doi: 10.1002/bco2.70179 (PMC12960746; doi:10.1002/bco2.70179)
Supplement: Supplementary file 1 — Table S1. Descriptive parameters of all patients with urinary bladder cancer in NU Hospital Group between 2010 and 2024 stratified into groups regarding to time to TURBT and the relation to the implementation of SCP. Figures represent number of patients (% of the column) if not otherwise indicated. (IQR: interquartile range, SCP: standardized care pathway, TURB: Transurethral resection of tumour in urinary bladder, IVIT: intra‐vesical instillation therapy, SLR: second look resection, MDTC: multidisciplinary team conference, RT: radiation therapy) [file BCO2-7-e70179-s001.docx]

**Supplementary Table 1. Descriptive parameters of all patients with urinary bladder cancer in NU Hospital Group between 2010 and 2024 stratified into groups regarding to time to TURBT and the relation to the implementation of SCP. Figures represent number of patients (% of the column) if not otherwise indicated.** (IQR: interquartile range, SCP: standardized care pathway, TURB: Transurethral resection of tumor in urinary bladder, IVIT: intra-vesical instillation therapy, SLR: second look resection, MDTC: multidisciplinary team conference, RT: radiation therapy)

| Variable name | | Before SCP 2010-2015 | | | | Early SCP 2016-2019 | | | | | | | Late SCP 2020-2024 | | | | |
| --- | --- | --- | --- | --- | --- | --- | --- | --- | --- | --- | --- | --- | --- | --- | --- | --- | --- |
|  |  | 0–18 Days | >18 Days | p value | | 0–18 Days | | >18 Days | | p value | | | 0–18 Days | | >18 Days | | p value |
| No. patients | **(% of the row)** | 122 (27) | 333 | |  | | 242 (73) | | 88 | |  | 255 (56) | | 199 | |  | |
| Gender, n (%) | **Male** | 93 (76) | 264 (79) | | 0.483 | | 178 (74) | | 67 (76) | | 0.635 | 196 (77) | | 164 (82) | | 0.148 | |
|  | **Female** | 29 (24) | 69 (21) | |  | | 64 (26) | | 21 (24) | |  | 59 (23) | | 35 (18) | |  | |
| Age (years) | **Median, (IQR)** | 76 (66–84) | 75 (68–81) | | 0.712 | | 75 (69–81) | | 78 (71–85) | | 0.014 | 76 (70–81) | | 76 (69–82) | | 0.799 | |
| Admission modality | **Referral** | 57 (47) | 314 (94) | | <0.001 | | 196 (81) | | 77 (88) | | 0.341 | 211 (83) | | 180 (91) | | 0.025 | |
|  | **Emergency** | 57 (47) | 12 (4) | |  | | 34 (14) | | 9 (10) | |  | 25 (10) | | 7 (4) | |  | |
|  | **Others^a^** | 8 (7) | 7 (2) | |  | | 12 (5) | | 2 (2) | |  | 19 (8) | | 12 (6) | |  | |
| Reason for investigation | **Macroscopic haematuria** | 88 (72) | 282 (79) | | 0.142 | | 196 (81) | | 63 (72) | | 0.066 | 204 (80) | | 145 (73) | | 0.074 | |
|  | **Others^b^** | 34 (28) | 71 (21) | |  | | 46 (19) | | 25 (28) | |  | 51 (20) | | 54 (27) | |  | |
| Radiology | **Diagnostically** | 109 (89) | 299 (90) | | 0.89 | | 236 (98) | | 84 (96) | | 0.333 | 238 (93) | | 191 (96) | | 0.220 | |
| Number of tumors^c^ | **Single** | 82 (80) | 212 (67) | | 0.404 | | 141 (60) | | 56 (64) | | 0.475 | 157 (62) | | 135 (68) | | 0.166 | |
|  | **Multiple** | 34 (29) | 107 (34) | |  | | 94 (40) | | 31 (36) | |  | 98 (38) | | 64 (32) | |  | |
| Tumor size^c^ | **≤30 mm** | 36 (47) | 133 (60) | | 0.049 | | 134 (67) | | 47 (67) | | 0.983 | 182 (71) | | 149 (75 | | 0.405 | |
|  | **> 30 mm** | 41 (53) | 90 (40) | |  | | 66 (33) | | 23 (33) | |  | 73 (29) | | 50 (25) | |  | |
| Tumor grade, n (%) | **G1** | 15 (12) | 43 (13) | | 0.73 | | 26 (11) | | 14 (16) | | 0.368 | 21 (8) | | 23 (12) | | 0.374 | |
|  | **G2** | 66 (54) | 191 (57) | |  | | 120 (50) | | 38 (43) | |  | 131 (52) | | 92 (46) | |  | |
|  | **G3** | 41 (34) | 99 (30) | |  | | 96 (40) | | 36 (41) | |  | 103 (40) | | 84 (42) | |  | |
| T, n (%) | **TaG1–2** | 50 (41) | 141 (42) | | 0.002 | | 116 (48) | | 46 (52) | | 0.015 | 142 (56) | | 103 (52) | | 0.814 | |
|  | **TaG3, Tis, T1** | 26 (21) | 115 (35) | |  | | 77 (32) | | 15 (17) | |  | 65 (25) | | 54 (27) | |  | |
|  | **T2+** | 46 (38) | 77 (23) | |  | | 49 (20) | | 27 (31) | |  | 48 (19) | | 42 (21) | |  | |
| N, n (%) | **N+** | 2 (2) | 5 (2) | | 0.916 | | 6 (3) | | 4 (5) | | 0.333 | 11 (4) | | 11 (6) | | 0.55 | |
| M, n (%) | **M1** | 3 (3) | 6 (2) | | 0.656 | | 7 (3) | | 1(1) | | 0.359 | 6 (2) | | 10 (5) | | 0.125 | |
| IVIT, n (%) | **For eligible patients^d^** | 25 (57) | 116 (67) | | 0.204 | | 92 (70) | | 14 (38) | | 0.002 | 91 (67) | | 54 (59) | | 0.224 | |
| SLR, n (%) | **For T1^e^** | 11 (42) | 35 (34) | | 0.448 | | 40 (67) | | 10 (77) | | 0.470 | 29 (76) | | 24 (63) | | 0.212 | |
| MDTC, n (%) | **For T1+^e^** | 3 (4) | 1 (1) | | 0.039 | | 94 (86) | | 38 (95) | | 0.136 | 74 (86) | | 72 (90) | | 0.434 | |
| Cystectomy, n (%) | **For T2+^e^** | 14 (30) | 50 (65) | | <0.001 | | 27 (55) | | 10 (37) | | 0.132 | 20 (42) | | 9 (21) | | 0.040 | |
| Palliation | **After first TURB** | 24 (20) | 33 (7) | | <0.001 | | 18 (7) | | 10 (11) | | 0.258 | 21 (8) | | 14 (7) | | 0.638 | |

**a** “Others” includes lower urinary tract symptom, urinary tract infection, etc.

**b** “Others” includes incidental findings, patient-reported symptoms, perioperative findings, and other non-haematuria indications.

**c** Missing samples for tumor size: 155 in 2010–2015, 60 in 2016–2019 and 0 in 2020–2024.

**d** Eligibility for IVIT defined: TaG1–2 with multiplicity and/or tumor size >3 cm, TaG3, Tis or T1.

**e** Patients within timeframes included in corresponding analyses: SLR – 6m, MDTC – 9m and cystectomy -14m.
